# Providing School Meals to All Students Free of Charge during the COVID-19 Pandemic and Beyond: Challenges and Benefits Reported by School Foodservice Professionals in California

**DOI:** 10.3390/nu14183855

**Published:** 2022-09-17

**Authors:** Monica D. Zuercher, Juliana F. W. Cohen, Christina E. Hecht, Kenneth Hecht, Lorrene D. Ritchie, Wendi Gosliner

**Affiliations:** 1 Nutrition Policy Institute, Division of Agriculture and Natural Resources, University of California, Berkeley, CA 94704, USA; 2 Center for Health Inclusion, Research, and Practice (CHIRP), Department of Public Health and Nutrition, Merrimack College, North Andover, MA 01845, USA; 3 Department of Nutrition, Harvard T.H. Chan School of Public Health, Boston, MA 02115, USA

**Keywords:** universal school meals, breakfast, lunch, nutrition, children

## Abstract

Universal school meals (USM) have the potential to increase access to healthy food for millions of U.S. students. This study evaluated school food authorities’ (SFA) perspectives of federal USM in response to COVID-19 (school year (SY) 2021–22) and California’s upcoming USM policy in the SY 2022–23. In February 2022, all SFAs in California (*n* = 1116) were invited to complete an online survey. Descriptive statistics and logistic regression examining differences by school demographic characteristics were used. Five hundred and eighty-one SFAs completed the survey; 63% of them first implemented USM during the COVID-19 pandemic. Reported benefits included increased student meal participation (79.2%) and reduced stigma (39.7%). Top challenges included staffing (76.9%) and meal packaging/solid waste (67.4%). Nearly all SFAs reported pandemic-related challenges procuring the necessary types (88.9%) and amounts of foods (85.9%), and non-food supplies/equipment (82.6%). Over 40% reported that federal reimbursements were insufficient to cover costs. SFAs with <40% FRPM-eligible students and/or higher student enrollment reported more current challenges and future concerns than those with ≥40% FRPMs and lower student enrollment. The top resources requested to implement CA’s USM included additional facilities/equipment (83.8%), communications/marketing (76.1%), increasing meal participation (71.5%), and financial management (61.5%). Most California SFAs reported that implementing federal USM had the intended effect of feeding more children. This study’s findings may be useful to the several other U.S. states implementing universal school meals in the SY 2022–23, and to other states or countries considering adopting a USM policy in the future.

## 1. Introduction

In the United States, the National School Lunch Program (NSLP) and the School Breakfast Program (SBP) provide nutritionally balanced meals to children in kindergarten–12th grade attending public and private nonprofit schools [1,2]. Childhood and adolescence are critical stages for physical and cognitive development, and nutrition plays a key role in facilitating these processes [3]. Malnutrition at these stages can have important long- and short-term educational and health implications, from developmental to cardiometabolic functions [3,4,5], and dietary behaviors adopted in early life can be carried to adulthood [6,7]. School meals have the potential to increase students’ access to healthy foods, and to facilitate children’s healthy food practices, which can support lifelong health [4,5,8].

In the school year (SY) 2018–2019, the NSLP operated in nearly 100,000 schools nationwide (approximately 95% of public and charter schools in the nation), and on average, it provided lunches to 29.6 million children each school day [9]; the SBP operated in about 90,000 schools nationwide, and on average, it provided breakfasts to 14.8 million children each school day [10]. Prior to the COVID-19 pandemic, students qualified for free, reduced-price, or “paid” meals (though these meals also receive a small federal subsidy) depending on their household income. In the SY 2018–19, 74.1% of NSLP meals were served free or at a reduced price [11]. The percentage of students in the United States that participated in the NSLP in the SY 2018–19 was 58.2% [11]. As a comparison, according to an international evaluation of meal programs, 85 out of the 103 responding countries had a large-scale school feeding program in the SY 2018–19; the average coverage rate in the most recently completed school year was 24% of students [12].

School meals are a main source of food and nutrients, particularly for children from households with low incomes, thus helping them to reduce food insecurity and increase equity [4,13,14]. School meals have been associated with multiple benefits for students, including improved dietary intake, food security, and academic performance [4,6,13,14,15,16]. To expand upon these benefits, efforts have been undertaken in the United States to improve the nutritional quality of school meals over time and better align them with the Dietary Guidelines for Americans, the most recent federal legislative effort being the Healthy, Hunger-Free Kids Act (HHFKA) of 2010 [17]. Studies have shown that the nutritional quality of school lunches and breakfasts (measured by the Healthy Eating Index (HEI) 2010) increased since the HHFKA’s nutritional standards were implemented [16,18]. Between the SY 2009–10 and the SY 2014–15, the total HEI 2010 score for school lunches increased from 58% to 82% (100% represents complete alignment between the meals served and the federal Dietary Guidelines), and the score for school breakfasts increased from 50% to 71% [18]. As a result, foods provided at schools in the U.S. now have a higher nutritional quality on average than foods from any other source in students’ lives, including food from grocery stores (prepared at home) and restaurants [13,19].

Since Congress first debated establishing a National School Lunch Program in the 1940s, advocates have considered whether these nutritious meals should be available to all students free of charge [20]. Providing free meals to all students has been viewed as way to reduce the stigma associated with qualifying for free or reduced-price meals (FRPM), improve nutrition, support working parents, and reduce the administrative burden of school meals on the school food authorities (SFA) responsible for implementing the school meal programs (typically a school or school’s SFA). The Community Eligibility Provision (CEP), and Provisions 1, 2, and 3, are examples of efforts to expand access to school meal programs [21,22]. The CEP allows schools located in low-income areas to provide breakfast and lunch free of charge to all students without the need for families to submit a meal application [21]. After being phased in over three school years, starting in the SY 2011–12, the CEP was expanded nationwide in the SY 2014–15 [21]. The number of schools adopting the CEP rose from 14,184 schools in 2014, to 33,171 schools in 2020 [23]. Provisions 2 and 3 are alternative provisions to the normal requirements for the annual determinations of eligibility for FRPM and daily meal counts by type [22]. These alternative options have been beneficial in reducing the administrative burden for schools and families [21,23].

Since the emergence of the COVID-19 pandemic in 2020, with authority and funding given by Congress, the USDA Food and Nutrition Service established multiple Child Nutrition waivers nationwide, with the aim of giving schools flexibility to keep operating meal programs despite pandemic-related challenges [24,25,26]. These waivers allowed schools to alter the way the school meals were served in order to support students’ access to nutritious meals, whether they were attending school in person or virtually, thus helping to minimize potential exposure to the coronavirus, allowing schools to continue to offer school meals during unexpected school closures and during the summer, and supporting a successful school reopening in the SY 2021–22 [24,27,28]. Schools also were allowed to provide meals at no cost to all students, regardless of FRPM eligibility based on household income, through the Seamless Summer Option waiver. As a result, nearly all (98.3%) school breakfasts and lunches were served free of charge in the SY 2020–21 [11,26]. 

The COVID-19 pandemic raised awareness of food insecurity and the implementation of waivers showed that universal school meals (USM) were possible [29]. In July 2021, California became the first state in the nation to fund a USM program when the federal waivers ended [30]. Beginning in the SY 2022–23, Californian SFAs serving students in grades K–12 are required to make breakfasts and lunches available, free of charge, every school day for students requesting a meal, regardless of their FRPM eligibility [31]. The California USM program differs from the federal USM program offered by the USDA through the Seamless Summer Option waiver in a number of ways, including that it requires making both breakfast and lunch available to all students daily (which is not a requirement in the USM Federal program). Californian USM require schools to optimize the federal reimbursements for which they are eligible. The state is committed to funding the costs of providing meals to all students beyond federal reimbursements. Maine quickly followed California in committing to provide USM, and more recently, Vermont and Massachusetts also passed policy to provide school meals at no cost for all students for at least the 2022–2023 school year [32,33]. 

Although the evidence highlights multiple benefits of USM, diverse factors have been found to make the implementation of CEP (i.e., USM at the school or district level) easier or more difficult [34]. These factors include SFA size, region, proportion of students certified for free meals, food production costs, staff size and capacity, food transportation costs, and access to suppliers. For example, school SFAs of a small size, located in rural areas, and with a low proportion of students certified for free meals, have been found to have more difficulties implementing USM via the CEP mechanism and implementing school meal programs in general [34,35].

Further study of USM programs is imperative to ensure that they are implemented optimally, that the effects of the programs are more completely understood, and that lessons learned inform other states considering such policy. The aim of this study was to assess Californian SFAs’ perspectives about the benefits and challenges experienced during the federally funded USM in response to the COVID-19 pandemic (SYs 2020–2022), and to identify anticipated concerns once California’s USM policy goes into effect in the SY 2022–23. We hypothesized that school food authorities with a smaller percentage of low-income students would face greater challenges, and that school district size and geographic characteristics (rural/urban) may be related to challenge, though assessing the direction of that relationship was exploratory.

## 2. Materials and Methods

### 2.1. Participants

On 7 February 2022, the California Department of Education circulated an initial email to all Californian SFAs (*n* = 1116) participating in the NSLP, inviting them to complete an online survey sharing their perspectives about providing school meals during the school year (SY 2021–22) when the federal COVID-19 waivers were in place, and during the next school year (SY 2022–23), when nearly all federal waivers terminate and California’s USM policy would go into effect. Two reminder emails were sent to elicit more responses; in total, the survey link was open for 4 weeks. Participation in the survey was voluntary and participants did not receive any incentives for participating.

### 2.2. Survey

The survey included 55 questions adapted from previous instruments [36,37,38,39,40,41,42,43], and it took participants approximately 30–60 min to complete (see Supplemental Materials). Survey domains included experiences implementing USM in the SY 2021–22, barriers to student meal participation, COVID-19 related supply chain issues, and anticipated concerns with USM during the SY 2022–23. Questions regarding changes due to USM in the SY 2021–22 were restricted to SFAs whose schools were not already providing free school meals to all students through CEP or another provision before the SY 2021–22 (*n* = 360). The survey was programmed and administered online using Qualtrics (Version March 2022, Provo, UT).

Most survey questions were multiple-choice, with the answers provided in a Likert scale order. Subsequently, those variables were dichotomized for analytical purposes (Table 1).

**Figure 1:**
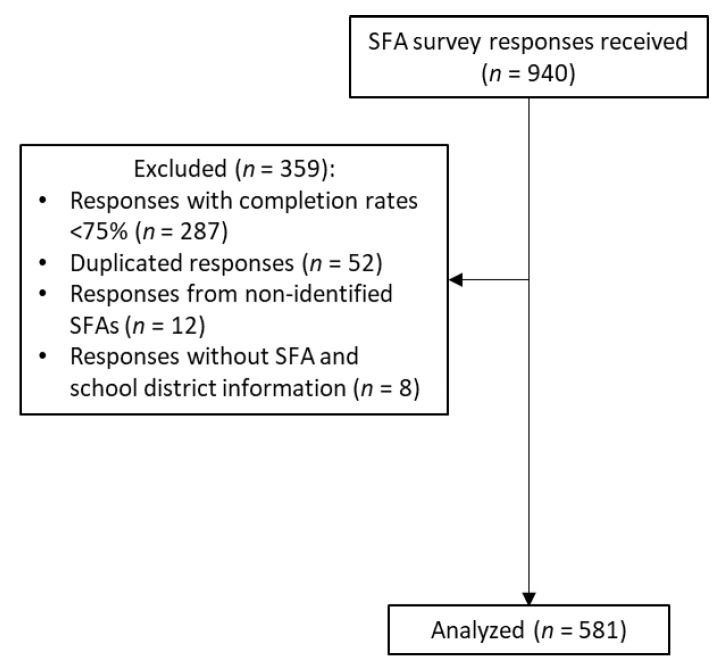
Sample size for the 2022 Californian school food authority survey.

A total of 940 surveys were received. Surveys with less than a 75% completion rate were excluded (*n* = 287) (completion rate was calculated based on the total number of questions the participant answered, and the logic conditioning of the questions was considered). Information regarding the total number of students and the percentage of students eligible for FRPMs in each SFA was obtained from the California Department of Education databases if missing from the survey response [44,45]. When a replicate response was identified (completed either by the same person more than one time or by different personnel from the same SFA), the most complete survey was used, and the other response(s) was removed from the analytical database (*n* = 52). Responses without any information about the represented SFA were removed (*n* = 8). Responses from SFAs not recognized by the California Department of Education, or schools that were part of a larger SFA, were also removed (*n* = 12) (Figure 1).

### 2.3. Stratification Variables

Results were stratified by the percentage of students qualifying for FRPM, by rural–urban classification, and by SFA size, based on the number of students. Stratification variables were selected based on prior studies of CEP [34]. For the FRPM classification, SFAs with 40% or fewer FRPM students were compared with SFAs with more than 40% FRPM in the SY 2021–22, based on data from the California Department of Education [45]. FRPM at 40% was used as it is similar to the cutoff required for CEP participation among schools in an SFA (≥40% of students directly certified as eligible for free school meals based on other means-tested programs such as SNAP). The urbanicity classification was made using the zip code reported by each SFA in the survey and based on the USDA rural–urban commuting area (RUCA) codes that classify U.S. census tracts using measures of population density, urbanization, and daily commuting [46,47]. Urban areas (RUCA primary code = 1) were compared with non-urban areas (RUCA primary codes = 2–10). SFA size classification was based on the total number of students in the SY 2021–22, as reported by the SFAs in the survey and from data from the California Department of Education [45]. Each SFA was categorized as: small = 2499 or less students; medium = 2500 to 9999 students; or large = 10,000 or more students. This is similar to the categories of student enrollment used in the USDA’s professional standards [48].

### 2.4. Statistical Analysis

Frequencies and percentages were used to describe categorical variables. In stratified analyses, logistic regression models were used to examine differences between FRPM eligibility, urbanicity, and size. Adjusted percentages and adjusted *p*-values were reported. The logistic regression models were adjusted by CEP status to account for those schools that were already providing free school meals to all students before the SY 2021–22, and urbanicity models were also adjusted for size. For pair-wise comparisons between small, medium, and large-sized SFAs, a Bonferroni correction was used to account for multiple comparisons (Bonferroni α = 0.025). All statistical analyses were conducted using Stata (StataCorp. 2021. Stata Statistical Software: Release 17. College Station, TX, USA).

## 3. Results

### 3.1. Sample Characteristics

The analytical sample included 581 SFAs representing 52.1% of the SFAs in California. Of those who responded to the survey, most were foodservice directors (67.3%), had been in their job for five years or more (53.1%), and more than half had a bachelor’s degree or higher (52.7%) (Table 2). Just over one third (37.0%) reported providing free school meals to all students before the SY 2021–22, through CEP or another provision. Most respondents represented small SFAs (53.2%), SFAs with more than 40% of students eligible for FRPM (70.4%), and SFAs in urban areas (66.3%). Nearly all (96.7%) SFAs implemented at least one COVID-19 waiver (mean implementation 7.1 ± 3.2 waivers).

### 3.2. Changes after Implementing USM during the COVID-19 Pandemic in the SY 2021–22

**Figure 2:**
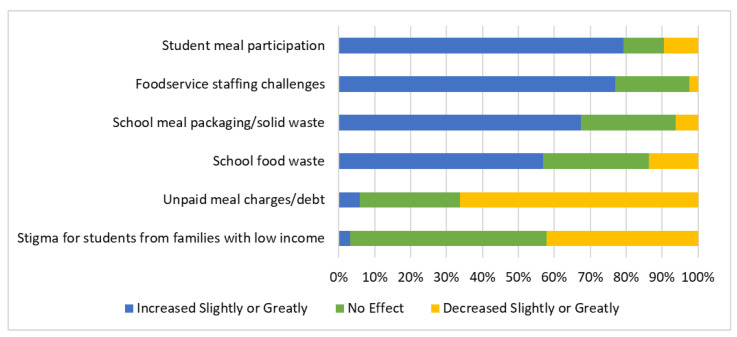
Changes reported after implementing universal school meals in the SY 2021–22 by Californian school food authorities (*n* = 360 to 359 depending on missingness of responses; excluded SFAs implementing universal school meals to all schools through CEP or another provision).

SFAs not previously implementing USM at all schools through CEP or another provision (*n* = 360) were asked to rate changes they attributed to offering USM during the pandemic in the SY 2021–22. Most reported experiencing increases (either slightly or greatly) in student meal participation (79.2% of SFAs), foodservice staffing challenges (76.9%), meal packaging/solid waste (67.4%), and food waste (56.8%). Many reported reductions (slightly or greatly) in unpaid meal charges (66.4%) and student stigma (45.7%) (Figure 2). The factors that SFAs reported to change the least included meal preparation using scratch/modified scratch cooking (51.3% reported “no effect”), amount of crowding in student dining areas (49%), and use of general funds to support school meal programs (48.4%) (Supplementary Table S1). Similar benefits and challenges were reported by SFAs that provided USM prior to the SY 2021–22 district-wide via other provisions such as CEP (Supplementary Figure S1).

Stratified analyses among SFAs not previously implementing USM in all schools through CEP or another provision showed that increases in student meal participation, foodservice staffing challenges, and waste (both school meal packaging/solid and food waste) were higher among SFAs with 40% or fewer students eligible for FRPM than in SFAs with more than 40% of students eligible for FRPM (*p* ≤ 0.05); no differences in FRPM eligibility were observed in terms of a reduction in stigma for students from families with a low income, or in unpaid meal charges/debt (*p* = 0.26 and *p* = 0.99, respectively) (Table 3). Increases in student meal participation were higher among non-urban SFAs than in urban SFAs (*p* = 0.046). SFAs with medium and large sizes had more foodservice staffing challenges than small SFAs (*p*-value for pairwise comparisons < 0.02). Medium and large SFAs also had a higher reported reduction in terms of stigma for students from families with a low income, and in unpaid meal charges/debt, than small SFAs (*p*-value for pairwise comparisons < 0.025).

### 3.3. Challenges Implementing USM during the COVID-19 Pandemic in the SY 2021–22

**Figure 3:**
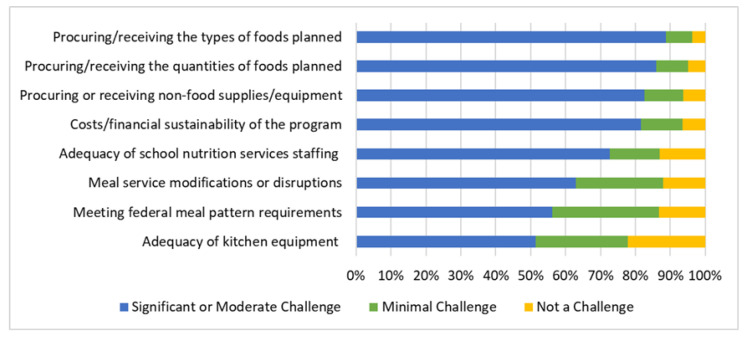
Challenges implementing universal school meals in the SY 2021–22, as per the Californian school food authorities (*n* = 577 to 575 depending on missingness of responses).

All SFAs were asked about challenges experienced implementing USM during the SY 2021–22. The greatest challenges reported by SFAs included the procurement of types of foods (88.9% reported this to be a significant or moderate challenge), amounts of foods (85.9%), non-food supplies/equipment (82.6%), financial sustainability of school meal programs (81.6%), and adequacy of school nutrition service staffing (72.6%) (Figure 3). Additional challenges reported as significant/moderate by most respondents included meal service modifications or disruptions (63.0%), meeting federal meal pattern requirements (56.3%), and adequacy of kitchen equipment (51.5%). The challenges that were least frequently reported to be significant/moderate included meeting student cultural food preferences (39.8%), reduced meal program participation (38.0%), meeting meal modifications for children with medically related food/nutrition needs (33.6%), and negative feedback or complaints about school meals from parents or students (26.4%) (Supplementary Table S2).

Stratified analyses showed there were no statistically significant differences in challenges experienced by FRPM eligibility or urbanicity (*p* > 0.05) (Table 4). SFAs with medium and large sizes perceived all the challenges as greater, compared with the perceptions of smaller SFAs (<0.025), except for meeting federal meal pattern requirements, a difference that was greater for larger rather than smaller SFAs, but it was not statistically different between small and medium SFAs (*p*-value for pairwise comparisons = 0.04 which is not smaller than the Bonferroni corrected value of 0.025).

### 3.4. Meal Reimbursements Relative to Costs of Implementing USM during the COVID-19 Pandemic in the SY 2021–22

**Figure 4:**
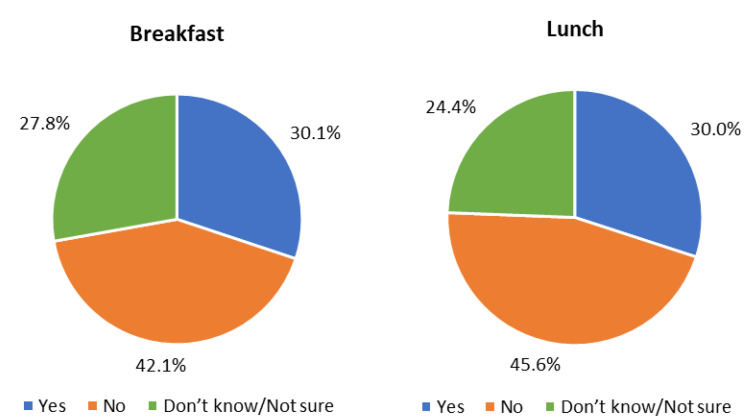
Responses by Californian school food authorities to the survey question: “Is the current meal reimbursement sufficient to cover the full cost of producing meals?” (*n* = 572 for breakfast *n* = 574 for lunch; response options included “yes”, “no”, and “don’t know/not sure”).

When SFAs were asked whether the current meal reimbursement was sufficient to cover the full cost of producing meals, fewer than a third said “yes” (Figure 4). Of those who said “no,” 37.9% said the current meal reimbursement covered 50% or less of the full cost of producing meals, about 41% said the current meal reimbursement covered between 51–75%, and about 20% said that the current meal reimbursement covered between 76–99% of the full cost. The average minimum per meal reimbursement rate SFAs reported in order to provide meals that meet all federal nutrition standards while appealing to students was USD 3.39 for breakfast and USD 5.08 for lunch. The average minimum per meal reimbursement rate SFAs reported in order to provide meals that regularly include fresh, locally grown produce was USD 3.75 for breakfast and USD 5.48 for lunch. The federal reimbursement rates during the SY 2021–22 were USD 2.4625 for breakfast and USD 4.3175 for lunch [49].

Stratified analyses found no statistically significant differences between the percentage of SFAs that reported that the current meal reimbursement is sufficient to cover the full cost of producing school breakfast and urbanicity, size, and FRPM eligibility (Supplementary Table S3). SFAs with a higher proportion of students eligible for FRPMs (>40%) reported that the current reimbursement is enough to cover the cost of producing school lunch more often than those with lower FRPM eligibility (43.3% vs. 31.0, respectively; *p* = 0.02). There was no statistically significant difference by urbanicity or size that was related to the percentage of SFAs that reported that the current meal reimbursement is not sufficient to cover the full cost of producing school lunches (*p* > 0.05).

### 3.5. Factors Driving a Financial Deficit for SFAs during the COVID-19 Pandemic in the SY 2021–22

**Figure 5:**
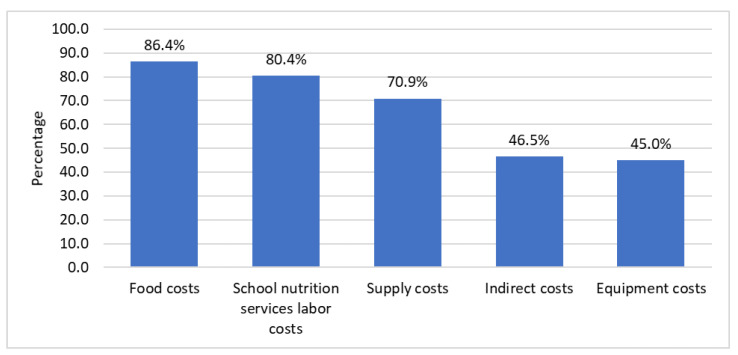
Main factors reported in driving the foodservice deficit for Californian school food authorities in the SY 2021–22 (*n* = 271).

Among SFAs that reported having a financial deficit in the SY 2021–22 (*n* = 271), the main factors driving that deficit included food costs (86.4%), labor relating to school nutritional services (80.4%), supply costs (70.9%), indirect costs (46.5%), and equipment costs (45.0%) (Figure 5). The least reported factors were increased meal program participation (14.4%), storage costs (17.3%), and facility costs (19.9%) (Supplementary Table S4).

Stratified analyses showed SFAs with more than 40% of their students eligible for FRPMs reported that their finances were more affected by supply costs than SFAs with 40% or fewer eligible FRPM students (*p* = 0.03) (Table 5). SFAs in non-urban areas reported that their finances were more affected by food costs and supply costs than SFAs in urban areas (*p* < 0.05). Medium and large SFAs reported that their finances were more affected by supply costs in comparison with small SFAs (*p*-value for pairwise comparisons < 0.025).

### 3.6. Concerns about the Future Operation of California’s USM

**Figure 6:**
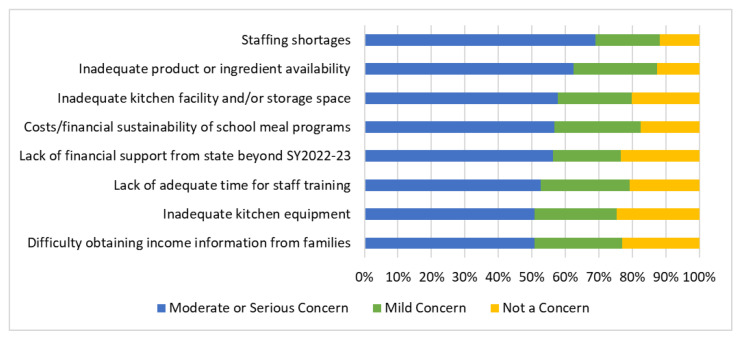
Greatest concerns about implementing universal school meals in the SY 2022–23 for Californian school food authorities (*n* = 578 to 574 depending on missingness of responses).

When asked about concerns related to implementing California’s USM, which goes into effect in the SY 2022–23, the major concerns reported by SFAs were related to COVID-19 rather than providing breakfast and lunch to all students free of charge, such as staffing shortages (69%) and inadequate product/ingredient availability (62.4%) (Figure 6). Concerns associated with infrastructure and staff skills included inadequate kitchen facilities/storage space (57.6%), lack of adequate time for staff training (52.6%), and inadequate kitchen equipment (50.8%). Financial concerns included the financial sustainability of school meal programs (56.7%), worries about a potential lack of financial support from the state for USM beyond the SY 2022–23 (56.2%), and difficulty obtaining income information from families (50.7%). The concerns that were reported as moderate or serious were the least common, such as low breakfast/ lunch participation (35.9% and 32.1%, respectively), loss of revenue from paid meals (29.7%), high breakfast/lunch participation (21.4% and 26%, respectively), and loss of revenue from competitive food and beverage sales (20.9%) (Supplementary Table S5).

Stratified analyses showed that concerns about staffing shortages, inadequate kitchen facilities and/or storage space, and inadequate kitchen equipment, were higher among SFAs with 40% or fewer students eligible for FRPMs than in those with more than 40% of students eligible for FRPMs (*p* ≤ 0.05) (Table 6). Concerns about implementing USM in the SY 2022–23 did not differ by urbanicity (*p* > 0.05). Large SFAs were more concerned about costs/financial sustainability of school meal programs and lack of financial support from the state for USM beyond the SY 2022–23 than small SFAs (*p* < 0.02). Large and medium SFAs were more concerned about staffing shortages, inadequate product or ingredient availability, inadequate kitchen facilities and/or storage space, lack of adequate time for staff training, and inadequate kitchen equipment, compared with small SFAs (*p*-value for pairwise comparisons < 0.025).

### 3.7. Additional Resources or Information Needed to Implement California’s USM in the SY 2022–23

**Figure 7:**
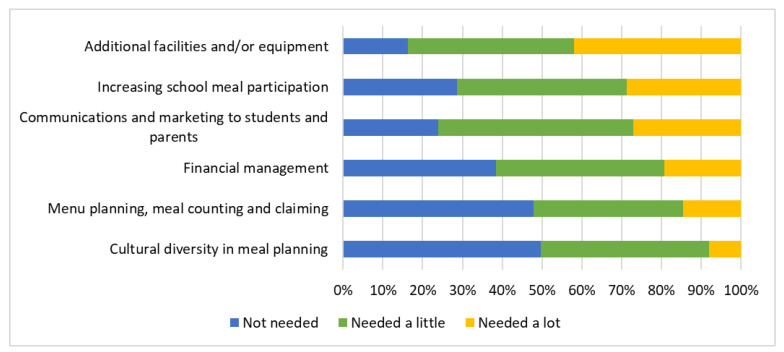
Additional resources or information needed by the Californian school food authorities to implement universal school meals in the SY 2022–23 (*n* = 572 to 577 depending on missingness of responses).

When SFAs were asked about the additional resources or information needed to implement CA’s USM in the SY 2022–23, the most common needs included additional facilities and/or equipment (reported as being needed “a little” or “a lot” by 83.8% of SFAs), communications and marketing to students and parents (76.1%), increasing school meal participation (71.5%), financial management (61.5%), menu planning, meal counting and claiming (52.2%), and cultural diversity in meal planning (50.4%) (Figure 7). The least reported needs included meeting special dietary needs (46.6%), food safety (37.5%), and making school meals more appealing to students (35.4%) (Supplementary Table S6).

Stratified analyses showed that SFAs with more than 40% of students eligible for FRPMs reported that communications and marketing to students and parents, and increasing school meal participation, were needed more often in this group than SFAs with 40% or fewer students eligible for FRPMs (*p* < 0.05) (Table 7). The additional resources or information needed for the SY 2022–23 did not differ by urbanicity (*p* > 0.05). Medium and large SFAs reported cultural diversity in meal planning as a need more often than small SFAs (*p*-value for pairwise comparisons < 0.025). Medium-sized SFAs reported additional facilities and/or equipment and increasing school meal participation as a need more often than small SFAs (*p*-value for pairwise comparisons < 0.025).

## 4. Discussion

Californian SFAs reported important benefits relating to providing USM using the federal SSO waivers during the SY 2021–22; namely, greater student meal participation, fewer unpaid meal charges, and reduced student stigma. Similar to these findings, a national study found that 85% of SFAs reported that serving USM during the pandemic eliminated stigma, and 81% reported that it eliminated school meal debt [26]. Contrary to Californian SFAs reporting increased school meal participation, nationally, in the SY 2019-20, the number of children participating in the school lunch program decreased by 24.3% compared with the SY 2018–19 [11]. These decreases in school meal participation may be due to the initial effects of the pandemic on reducing access to school meals during remote learning days [50]. It will be important to monitor the impact of USM on school meal participation in California and other states continuing to offer USM as COVID-related absences from school diminish.

In the present study, increased school meal participation was more often reported by SFAs with 40% or fewer students eligible for FRPM. Given the expansion of CEP nationally, and because most students participating in the school meal programs have been deemed as FRPM-eligible (U.S. Department of Agriculture, 2022b), schools with fewer FRPM-eligible students had the most room for improving the uptake of school meals. Other studies have similarly shown that when school meals were offered free of charge to all students through CEP, the greatest increase in participation was among students from families not eligible for FRPM [4], and this increased participation occurred particularly among students near or above the eligibility cutoff for FRPM [8]. Given the current U.S. context, wherein over 60% of adults suffer from a diet-related disease [51], the average diet quality of children is marginal [5], and school meals reduce diet-related disparities [13] and are the healthiest options consumed by most children [19], increasing the uptake of school meals by all students remains a laudable public health goal. Furthermore, even if a smaller percentage of FRPM-eligible students are new participants in school meals when they become universally available, those students may receive a disproportionate benefit from their participation, improving food security, reducing stigma, and thus potentially improving wellbeing, learning, and academic success. A recent estimate suggested that school meal participation at rates prior to USM implementation could result in estimated savings of USD 21 bn annually from improvements to child food security and health [52]; thus, increasing the student uptake of school meals would potentially increase savings.

Californian SFAs reported that meal packaging and food waste increased as a result of providing USM in the SY 2021–22. Similar findings regarding solid waste have been reported in another study that evaluated school meals during COVID-19, an increase attributed to the greater use of prepackaged and individual servings in order to mitigate infection transmission [53]. More students eating school meals translates into greater overall packaging and food waste in the absence of waste mitigation efforts. A recent study estimated that USD 1.7 bn of school food is wasted annually [54]. Future studies should examine whether the per student waste, generated by school food services, is impacted by the implementation of USM outside of COVID-19 conditions, and by adding an extra meal per day (for those schools that were not offering breakfast but they are going to be required to make it available to students in the SY 2022–23). Regardless, efforts to reduce school food waste are needed, and understanding how this waste can be reduced while increasing student meal participation will be especially important.

Most challenges concerning the implementation of USM through the USDA’s federal program, as reported by California SFAs, were associated with supply-chain and staffing issues. Similarly, other studies reported that most school districts were experiencing supply-chain issues (reported by > 92%), limited product availability (>80%), and labor shortages (73%) which were associated with school meal operations during the pandemic [26,38]. It is anticipated that the implementation of USM in states such as California, which will continue to offer them after the federal program expires, will become less challenging as the economic fallout and other disruptions from the pandemic resolve. In our study, Californian SFAs requested additional resources to implement the state’s USM program in the SY 2022–23 to address their need for additional facilities and/or equipment and financial assistance.

During the COVID-19 pandemic in the SY 2021–22, SFAs nationwide were able to offer USM through the Seamless Summer Option waiver (SSO), which allowed the provision of free school meals to all students at higher Summer Food Service Program (SFSP) reimbursement rates. Over 90% of the study sample implemented the SSO Program waiver (with SFSP reimbursement rates of USD 4.3175 per lunch and USD 2.4625 per breakfast) [55]. Moreover, Californian SFAs relied heavily upon USDA waivers to operate during the COVID-19 pandemic. Meal Pattern, Non-Congregate Feeding, and Meal Time waivers allowing flexibilities in what, how, and when meals could be served were also commonly adopted [26,38]. Despite the use of waivers, many SFAs experienced challenges, thus highlighting the importance of continuing flexibilities until the supply-chain and other pandemic-related issues resolve. 

Californian SFAs reported requiring an additional average of USD 0.93 per breakfast and USD 0.77 per lunch to serve meals that meet all federal nutrition standards and appeal to students. Between 42–45% reported that the current reimbursement rate was not enough to cover the cost of producing school meals, which is higher than the results from a national SFA survey conducted in 2021 [38]. In a national study, smaller SFAs were more likely to report a deficit (defined as costs that exceed revenues), whereas in our California sample, large and medium-sized SFAs were more likely to report a deficit (defined as the meal reimbursement not being enough to cover the full cost of producing meals) [38]. Prior research conducted before the pandemic has shown that implementing USM through CEP reduced costs for school lunch (by USD 0.63) and breakfast (by USD 0.58) among large and medium-sized schools, but not among smaller schools, and that these cost savings were not associated with reducing the nutritional quality of the meals [56]. Finally, it is important to acknowledge that the Californian SFAs reported on the financial sustainability of USM during a time of rising food, labor, and supply costs [57,58]. It will be important to evaluate whether, and how, the challenges, concerns, and costs of USM change under the new waivers and after all flexibilities are lifted.

It is important to highlight that several funds, resources, and waiver extensions have been made available to SFAs after study data were collected. Passage of the federal Keep Kids Fed Act of 2022 in June 2022 extended some waiver flexibilities, notably the supply chain disruption waiver for meal pattern violations for the SY 2022–23 [59,60,61,62]. The Act also increased the federal reimbursement rate for school lunch by USD 0.40 and for school breakfast by USD 0.15 for the SY 2022–23 [60]. Together with USDA’s required annual adjustment in reimbursement, this resulted in increases in the federal meal reimbursement rate of approximately USD 0.68 per free/reduced-price lunch and USD 0.32 per free/reduced price breakfast for the SY 2022–23 [63]. Furthermore, California has funded a variety of complementary supports to USM, including funds to upgrade school kitchen infrastructure and equipment to incorporate more fresh, minimally processed Californian-grown foods in school meals (USD 450 million available over three years), and to provide additional training and technical assistance (USD 100 million); funds to support the School Breakfast and Summer Meal Start-Up and Expansion Grant Program (USD 3 million); and to support the California Farm to School Program (USD 60 million over two years) [64,65,66,67].

Among Californian SFAs, more concerns and challenges were reported by SFAs with 40% or fewer students eligible for FRPM compared with those that had 40% or more students eligible for FRPM, and among larger and medium-sized SFAs (≥2500 students) compared with smaller SFAs. In a national study, larger and rural SFAs were more likely to report challenges with supply chain disruptions than their smaller and non-rural counterparts [38]. Schools with fewer FRPM-eligible students may have experienced more challenges as their student participation in school meals increased further. Few differences were observed in our study based on urbanicity; however, most SFAs in our California sample were in urban areas and only 6.5% were in the most rural classification (RUCA codes 8–10). 

USM have the potential to increase access to healthy food for millions of U.S. students, but in order to make these programs successful, it is important to recognize that there are issues to be addressed. As mentioned earlier in this discussion, several efforts have been made by the federal government and the state of California to address some of the staffing, financial, and infrastructure issues that SFAs faced, while offering free school meals for all in the SY 2020–22. Regarding the increase in meal packaging and food waste, which was one of the changes reported by many SFAs, previous studies have proposed strategies that could be implemented to increase meal consumption, and consequently, reduce food waste and increase variety in school meals, offer more culturally appropriate foods that represent the student population, give students enough time to eat, and limit students’ access to competitive foods during the school day, among others [68]. In addition to those strategies, a reduction in the use of prepacked and individually wrapped foods, as well as disposable containers and utensils, could also help reduce meal packaging and solid waste.

### Strengths and Limitations

Strengths of this study include a high response rate with a sample representative of SFAs in the state of California regarding FRPM eligibility and CEP status. For context, in the state of California, 72.5% of schools have a FRPM program > 40% (compared with 70.4% in the study sample) and 34.5% participate in CEP (compared with 37.0% in the sample) [38,69]. One study limitation is that the sample was not necessarily representative of SFAs in the state regarding urbanicity and size. The percentage of large SFAs in our study sample was 19.2% versus 13.0% in the state, and 66.3% were located in urban areas compared with 40.9% in the state (information provided by the California Department of Education). Moreover, SFAs were surveyed at a single point of time in one state, thus reducing generalizability regarding the benefits and challenges of USM in other contexts (e.g., in the absence of recovery from a pandemic). All data were collected via self-reports. It is possible that SFAs that had more or fewer challenges to report were more likely to volunteer to complete the survey. It was made clear to SFAs that their individual responses would remain anonymous and that University of California researchers would access and analyze the data; however, it is possible that SFA responses were biased given the fact that the California Department of Education, the state agency that oversees and monitors the school meal programs, sent the survey invitation. 

Finally, we highlight that this study sought to primarily understand the benefits and challenges of implementing the USM federal program during the COVID-19 pandemic in the SY 2021–22, in order to inform the initiation of California’s USM. The federal USM program was profoundly affected by the repercussions of the COVID-19 pandemic, including the impacts on staffing, availability of food, and other supplies, as well as impacts on storage/operations and finances. For example, the COVID-19 pandemic affected storage and operations because SFAs needed to use and store more disposable service-ware and personal protective equipment (PPE), implement intensified cleaning schedules, and they experienced a reduced capacity for typical food service models due to distancing requirements. Finances were negatively affected by the purchases of PPE and disposable service-ware, increased food costs, and supply shortages requiring non-bulk or retail purchases. In summary, the conditions in which the federal USM program was implemented, as well as the structure of the program itself, are different from the conditions that the CA USM program will encounter; thus, ongoing study of USM will be essential. The present study investigated anticipated barriers and challenges to implementing California’s USM program that takes effect in the SY 2022–23, in which schools will be required to offer both breakfast and lunch daily to all students. Of note, most SFAs did not express concerns about providing meals to all students free of charge; the anticipated challenges focused much more on the expectation that supply chain and labor issues experienced during the COVID-19 pandemic would continue into the 2022–2023 school year. California’s substantial commitment of new resources and funding to improve access to, and the quality of, school meals will address many of the challenges SFAs reported as concerns. Understanding how those resources impact the SFAs’ ability to meet students’ needs and provide healthy, appealing meals to all students free of charge will provide important insights to make the California’s USM program and school meal programs nationwide more successful.

## 5. Conclusions

California school food authorities report that providing school meals to all students free of charge was beneficial in increasing meal participation and reducing stigma and unpaid meal charges; these outcomes are consistent with the objectives of USM in terms of addressing nutrition equity and food security during the COVID-19 pandemic. However, these improvements were not without challenges related to the pandemic; namely, supply chain disruptions and staffing inadequacies, and challenges related to providing more school meals to students, such as inadequate kitchen facilities and increased solid food waste. School meal programs have the potential to provide healthy foods and beverages, and a model of healthy eating, to most of the child population. Extensive research has documented the numerous benefits of free school meals for students and their families [4]. Identifying the most promising practices for feeding all students, while managing labor, supply chain, and waste issues, will be critical to successful school meal implementation in California, and for informing other states or countries that are considering adopting USM policy in the future.

## Figures and Tables

**Table 1:** Description of original and operational version of the variables.

Survey Domain	Answer Choices in the Survey	Analytical Version
Changes due to USM in the SY 2021–22: Survey question 28 *	“decreased greatly”, “decreased slightly”, “no effect”, “increased slightly”, and “increased greatly”	“increased slightly or greatly” vs. “decreased slightly or no effect”
Challenges implementing USM in the SY 2021–22: Survey question 27 *	“significant challenge”, “moderate challenge”, “minimal challenge”, and “not a challenge”	“moderate or significant challenge” vs. “minimal or not a challenge”
Concerns for the future of USM: Survey questions 30-32 *	“not a concern”, “mild concern”, “moderate concern”, and “serious concern”	“moderate or serious concern” vs. “mild or not a concern”
Additional resources or information needed for the SY 2022–23: Survey question 34 *	“not needed”, “needed a little”, and “needed a lot”	“needed a little or a lot” vs. “not needed”
Factors driving deficit in the SY 2021–22: Survey question 46 *	“yes” and “no”	“yes” vs. “no”

* Survey is available in Supplementary Survey.

**Table 2:** Characteristics of survey respondents and their California school food authorities (*n* = 581) ^1^.

**Survey Respondent Characteristics**	***n***	**%**
Title		
SFA Foodservice/School Nutrition Director	391	67.3
School Nutrition Supervisor/Manager	99	17.0
Other	91	15.7
Years in the role at SFA		
Less than 1 year	81	14.0
1–4 years	190	32.8
5–9 years	174	30.0
10 years or more	134	23.1
Years in other institutional, restaurant, or commercial foodservice		
0–4 years	291	50.3
5–14 years	120	20.7
15 or more years	168	29.0
Highest education		
High school or less	60	10.4
Some college, no degree	131	22.7
Associate degree	82	14.2
Bachelor’s degree	207	35.9
Master’s degree or more	97	16.8
**School Food Authority Characteristics in the SY 2021–22**	***n***	**%**
Provided universal school meals before the SY 2021–22 district-wide	211	37.0
Size		
Small (2499 or less students)	308	53.2
Medium (2500–9999 students)	160	27.6
Large (10,000 or more students	111	19.2
Free and reduced-price meal eligibility		
≤40% of students	172	29.6
>40% of students	409	70.4
Urban–rural classification		
Urban (RUCA primary code 1) ^2^	385	66.3
Not urban (RUCA primary codes 2–10)	196	33.7
**COVID-19 Waivers Implemented in the SY 2021–22 ^3^**	***n***	**%**
Seamless Summer Option	536	93.2
Meal Pattern	477	83.8
Meal Service Time Flexibility	411	72.9
Non-congregate Feeding	411	72.6
Parent and Guardian Pick-up	386	68.3
Offer Versus Serve	375	67.0
Monitoring Waivers	360	64.8
Local School Wellness Policy Triennial Assessments	353	63.4
Area Eligibility	234	42.7
Selected Reporting Requirements	224	40.7
Community Eligibility	177	31.8
Foodservice Management	97	17.7

^1^ Sample size varies for some questions due to missing survey responses. ^2^ RUCA codes: USDA rural–urban commuting area codes. ^3^ Description of the COVID-19 waivers can be found on the USDA website [24].

**Table 3:** Frequency of changes reported after implementing universal school meals in the SY 2021–22 using select characteristics noted by the Californian school food authorities (*n* = 360) ^1^.

Change	FRPM Eligibility ^2^			Urbanicity ^3^			Size ^4^			
	≤40% (*n* = 145)	>40% (*n* = 215)	*p*-Value	Urban (*n* = 254)	Non-Urban (*n* = 106)	*p*-Value	Large *(n* = 180)	Medium (*n* = 106)	Small (*n* = 174)	*p*-Value
	%			%			%			
**Changes that most reported as having increased in the SY 2021–22 ^5^**										
Student meal participation	90.1	72.0	0.0001	75.8	86.3	0.04	76.3	81.1	79.3	0.68
Foodservice staffing challenges	85.9	71.1	0.001	78.4	74.4	0.40	86.3	88.7	65.5	0.0001 ^a,b^
School meal packaging/solid waste	75.0	62.4	0.01	66.9	68.5	0.78	69.2	73.6	62.7	0.18
School food waste	66.9	50.2	0.002	58.6	52.9	0.36	63.8	51.4	56.9	0.47
**Changes that most reported as having decreased in the SY 2021–22 ^6^**										
Unpaid meal charges/debt	64.1	67.9	0.46	64.4	70.1	0.30	82.7	75.5	53.2	0.0001 ^a,b^
Stigma for students from families with low income	48.9	43.6	0.32	45.6	45.9	0.96	55.0	55.7	35.3	0.01 ^a,b^

^1^ Data is only for subset of sample (*n* = 360) that did not implement USM SFA-wide through CEP or another provision prior to the SY 2021–22. Sample size varies for some questions due to missing survey responses.^2^ Free and reduced-price meal (FRPM) eligibility was defined as SFAs with 40% or fewer FRPM students vs. SFAs with more than 40% FRPM students in the SY 2021–22. Unadjusted percentages and *p*-values were reported. ^3^ Urbanicity was defined using the USDA rural–urban commuting area (RUCA) codes as urban areas = RUCA primary code 1 and non-urban areas = RUCA primary codes 2–10. Models for urbanicity were adjusted for size. Adjusted percentages and *p*-values were reported. ^4^ SFA size was defined as small = 2499 or fewer students, medium = 2500 to 9999 students, and large = 10,000 or more students. *p*-values for size represent the overall effect of size. The statistical significance for pairwise comparisons is indicated as follows: ^a^ differences between medium and small; ^b^ differences between large and small; no significant differences were observed between medium and large. Unadjusted percentages and *p*-values were reported. ^5^ Frequencies representing SFAs that identified the changes as having increased slightly or greatly. Other answer options were: “no effect”, “decreased slightly”, and “decreased greatly”. ^6^ Frequencies representing SFAs that identified the changes as having decreased slightly or greatly; other answer options were: “no effect”, “increased slightly”, and “increased greatly”.

**Table 4:** Frequency of challenges with regard to school meals experienced in the SY 2021–22, using select SFA characteristics noted by the Californian school food authorities (*n* = 581) ^1^.

Challenge ^2^	FRPM Eligibility ^3^			Urbanicity ^4^			Size ^5^			
	≤40% (*n* = 145)	>40% (*n* = 215)	*p*-Value	Urban (*n* = 254)	Non-Urban (*n* = 106)	*p*-Value	Large (*n* = 180)	Medium (*n* = 106)	Small (*n* = 174)	*p*-Value
	%			%			%			
Procuring or receiving the types of foods or beverages planned	87.0	89.9	0.36	89.9	88.3	0.55	97.3	96.2	82.4	0.0001 ^a,b^
Procuring or receiving the quantities of foods or beverages planned	82.3	87.3	0.15	85.1	87.0	0.51	97.4	95.6	76.4	0.0001 ^a,b^
Procuring or receiving non-food supplies or equipment needed for school meals	78.3	84.1	0.13	80.1	85.7	0.08	98.2	92.3	71.8	0.0001 ^a,b^
Costs/financial sustainability of school meal programs	84.3	80.5	0.30	82.6	80.4	0.54	88.5	86.1	76.8	0.003 ^a,b^
Adequacy of school nutrition services staffing	76.3	72.2	0.35	73.8	72.8	0.79	89.6	83.3	62.5	0.0001 ^a,b^
Meal service modifications or disruptions	59.5	65.2	0.22	64.9	61.0	0.39	71.3	69.6	57.5	0.003 ^a,b^
Meeting federal meal pattern requirements	55.8	56.6	0.87	58.0	53.2	0.31	72.1	59.4	49.0	0.01 ^b^

^1^ Sample size varies for some questions due to missing survey responses. ^2^ Frequencies representing SFAs that identified the challenges as moderate or significant; other answer options were: “minimum challenge” and “not a challenge”. ^3^ Free and reduced-price meal (FRPM) eligibility was defined in terms of SFAs with 40% or fewer FRPM students vs. SFAs with more than 40% FRPM students in the SY 2021–22. Models for FRPM eligibility were adjusted by CEP status. Adjusted percentages and *p*-values were reported. ^4^ Urbanicity was defined using the USDA rural–urban commuting area (RUCA) codes as urban areas = RUCA primary code 1 and non-urban areas = RUCA primary codes 2–10. Models for urbanicity were adjusted by CEP status and size. Adjusted percentages and *p*-values were reported. ^5^ SFA size was defined as small = 2499 or fewer students, medium = 2500 to 9999 students, and large = 10,000 or more students. Models for the sizes represented were adjusted by CEP status and *p*-values represent the overall effect of size. Adjusted percentages and *p*-values were reported. The statistical significance for pairwise comparisons is indicated as follows: ^a^ differences between medium and small; ^b^ differences between large and small; no significant differences were observed between medium and large.

**Table 5:** Main factors reported in driving the foodservice deficit in the SY 2021–22 using the select characteristics noted by the Californian school food authorities (*n* = 271) ^1^.

Factor ^2^	FRPM Eligibility ^3^			Urbanicity ^4^			Size ^5^			
	≤40% (*n* = 145)	>40% (*n* = 215)	*p*-Value	Urban (*n* = 254)	Non-Urban (*n* = 106)	*p*-Value	Large (*n* = 180)	Medium (*n* = 106)	Small (*n* = 174)	*p*-Value
	%			%			%			
Food costs	83.0	87.9	0.30	82.3	93.1	0.02	91.7	87.1	83.5	0.50
School nutrition services’ labor costs	77.6	81.5	0.50	76.7	86.2	0.07	87.9	84.4	75.4	0.14
Supply costs	61.7	75.3	0.03	63.8	82.9	0.001	78.7	83.3	60.6	0.001 ^a,b^
Indirect costs	46.4	46.8	0.96	44.6	50.6	0.40	58.7	51.5	38.8	0.09
Equipment costs	50.7	42.5	0.23	42.6	51.5	0.22	43.3	52.1	42.9	0.21

^1^ Data is only for subset of sample (*n* = 271) that reported having a deficit. Sample size varies for some questions due to missing survey responses. ^2^ Frequencies representing SFAs that reported factors that were driving the foodservice deficit; answer options were: “yes” and “no”. ^3^ Free and reduced-price meal (FRPM) eligibility was defined in terms of SFAs with 40% or fewer FRPM students vs. SFAs with more than 40% FRPM students in the SY 2021–22. Models for FRPM eligibility were adjusted by CEP status. Adjusted percentages and *p*-values were reported. ^4^ Urbanicity was defined using the USDA rural–urban commuting area (RUCA) codes as urban areas = RUCA primary code 1 and non-urban areas = RUCA primary codes 2–10. Models for urbanicity were adjusted by CEP status and size. Adjusted percentages and *p*-values were reported. ^5^ SFA size was defined as small = 2499 or fewer students, medium = 2500 to 9999 students, and large = 10,000 or more students. Models for size were adjusted by CEP status and *p*-values represent the overall effect of size. Adjusted percentages and *p*-values were reported. The statistical significance for pairwise comparisons is indicated as follow: ^a^ differences between medium and small; ^b^ differences between large and small; no significant differences were observed between medium and large.

**Table 6:** Greatest concerns about implementing California’s universal school meals in SY2022–23 by select characteristics of school food authorities (*n* = 581) ^1^.

Concerns ^2^	FRPM Eligibility ^3^			Urbanicity ^4^			Size ^5^			
	≤40% (*n* = 145)	>40% (*n* = 215)	*p*-Value	Urban (*n* = 254)	Non-Urban (*n* = 106)	*p*-Value	Large (*n* = 180)	Medium (*n* = 106)	Small (*n* = 174)	*p*-Value
	%			%			%			
Staffing shortages	77.1	65.8	0.01	69.5	68.3	0.77	84.8	83.3	55.9	0.0001 ^a,b^
Inadequate product or ingredient availability	63.4	63.0	0.94	61.6	65.8	0.32	83.2	76.3	48.9	0.0001 ^a,b^
Inadequate kitchen facilities and/or storage space	67.4	54.3	0.01	59.3	55.8	0.44	67.8	68.3	49.2	0.0001 ^a,b^
Costs/financial sustainability of school meal programs	60.7	54.6	0.19	57.2	54.7	0.59	63.8	59.2	52.2	0.02 ^b^
Lack of financial support from state for the Universal Meals Program beyond SY2022–23	60.9	54.5	0.18	55.8	57.5	0.72	67.1	55.6	52.9	0.02 ^b^
Lack of adequate time for staff training	59.8	50.3	0.05	51.4	56.4	0.28	62.1	65.6	43.1	0.0001 ^a,b^
Difficulty obtaining income information from families	52.8	49.9	0.55	51.9	48.6	0.49	54.3	48.2	50.9	0.69
Inadequate kitchen equipment	58.3	48.2	0.04	50.4	52.6	0.63	60.0	58.1	44.2	0.01 ^a,b^

^1^ Sample size varies for some questions due to missing survey responses. ^2^ Frequencies representing SFAs that identified the concern as moderate or serious; other answer options were: mild concern and not a concern. ^3^ Free and reduced-price meal (FRPM) eligibility was defined as SFAs with 40% or less FRPM students vs. SFAs with more than 40% FRPM students in the SY 2021–22. Models for FRPM eligibility were adjusted by CEP status. Adjusted percentages and *p*-values were reported. ^4^ Urbanicity was defined using the USDA rural–urban commuting area (RUCA) codes as urban areas = RUCA primary code 1 and non-urban areas = RUCA primary codes 2–10. Models for urbanicity were adjusted by CEP status and size. Adjusted percentages and *p*-values were reported. ^5^ SFA size was defined as small = 2499 or fewer students, medium = 2500 to 9999 students, and large = 10,000 or more students. Models for size were adjusted by CEP status and *p*-values represent the overall effect of size. Adjusted percentages and *p*-values were reported. The statistical significance for pairwise comparisons is indicated as follows: ^a^ differences between medium and small; ^b^ differences between large and small; no significant differences were observed between medium and large.

**Table 7:** Additional resources or information needed for the SY 2022–23 using characteristics noted by Californian school food authorities (*n* = 581) ^1^.

Resource ^2^	FRPM Eligibility ^3^			Urbanicity ^4^			Size ^5^			
	≤40% (*n* = 145)	>40% (*n* = 215)	*p*-Value	Urban (*n* = 254)	Non-Urban (*n* = 106)	*p*-Value	Large (*n* = 180)	Medium (*n* = 106)	Small (*n* = 174)	*p*-Value
	%			%			%			
Additional facilities and/or equipment	85.0	83.8	0.74	83.5	85.3	0.58	89.3	89.0	80.0	0.008 ^a^
Communications and marketing to students and parents	65.8	80.1	0.001	75.5	77.8	0.55	77.8	83.1	72.3	0.08
Increasing school meal participation	57.1	76.7	0.0001	68.6	76.3	0.07	75.1	78.5	66.1	0.02 ^a^
Fiscal management	60.8	62.4	0.75	61.3	63.8	0.59	59.2	68.3	60.0	0.72
Menu planning, meal counting and claiming	51.3	52.8	0.75	55.2	47.4	0.11	55.2	54.1	50.8	0.38
Cultural diversity in meal planning	49.3	51.2	0.69	51.4	49.1	0.64	59.3	58.0	43.5	0.01 ^a,b^

^1^ Sample size varies for some questions due to missing survey responses. ^2^ Frequencies representing SFAs that identified the resources as needed “a little” or “a lot”; other answer options were: “not needed”. ^3^ Free and reduced-price meal (FRPM) eligibility was defined as SFAs with 40% or less FRPM students vs. SFAs with more than 40% FRPM students in the SY 2021–22. Models for FRPM Eligibility were adjusted by CEP status. Adjusted percentages and *p*-values were reported. ^4^ Urbanicity was defined using the USDA rural–urban commuting area (RUCA) codes as urban areas = RUCA primary code 1 and non-urban areas = RUCA primary codes 2–10. Models for urbanicity were adjusted by CEP status and size. Adjusted percentages and *p*-values were reported. ^5^ SFA size was defined as small = 2499 or fewer students, medium = 2500 to 9999 students, and large = 10,000 or more students. Models for size were adjusted by CEP status and *p*-values represent the overall effect of size. Adjusted percentages and *p*-values were reported. The statistical significance for pairwise comparisons is indicated as follows: ^a^ differences between medium and small; ^b^ differences between large and small; no significant differences were observed between medium and large.

## Data Availability

The data are not publicly available as they are part of an ongoing evaluation that has not yet been completed.

## Supplementary Materials

The following supporting information can be downloaded at: https://www.mdpi.com/article/10.3390/nu14183855/s1, Figure S1: Benefits and challenges of USM among SFAs that provided universal school meals before the SY 2021–22 district-wide; Table S1: Percentage of changes reported after implementing universal school meals in the SY 2021–22 by Californian school food authorities; Table S2: Percentage of challenges implementing universal school meals during the COVID-19 pandemic in the SY 2021–22 by Californian school food authorities; Table S3: Percentage of SFAs that reported that the current meal reimbursement is sufficient to cover the full cost of producing school meals according to the select characteristics noted by Californian school food authorities; Table S4: Factors reported driving the foodservice deficit for Californian school food authorities during the COVID-19 pandemic in the SY 2021–22; Table S5: Percentage of concerns about implementing universal school meals in the SY 2022–23 for Californian school food authorities; Table S6: Percentage of additional resources or information needed by Californian school food authorities to implement universal school meals in the SY 2022–23.
