# Supplementary material for: Providing School Meals to All Students Free of Charge during the COVID-19 Pandemic and Beyond: Challenges and Benefits Reported by School Foodservice Professionals in California
Source: Nutrients. 2022 Sep 17;14(18):3855. doi: 10.3390/nu14183855 (PMC9500887; doi:10.3390/nu14183855)
Supplement: Supplementary file 1 [file nutrients-14-03855-s001.zip › Statewide Survey of FSDs CA.pdf]

Online Statewide Survey of Foodservice Directors

[Domain: introduction]

Thank you for taking part in this research about school meals. Your responses will help inform support for school meal operations next school year, when California rolls out the new Universal Meals Program. This survey will take approximately 30-60 minutes to complete.

We will combine survey results from multiple School Food Authorities and you and your school district will not be identified with your responses. Please answer all of the questions to the best of your knowledge. If you cannot answer a question, you can skip it, but we hope that you will answer all of the questions. Please note that questions on this survey may ask about different school years (for example, prior to the pandemic, during the pandemic, currently while the federal Seamless Summer Option (SSO) is allowed for reimbursable school meals, and in the future when the state's Universal Meals Program begins).

If you need to stop before completing the survey, you can return and continue where you left off at any time before February 22. If you have any questions, please contact Principal Investigator Wendi Gosliner at (510) 612-1552 or wgosliner@ucanr.edu.

Please ensure there is only one survey entry per school food authority.

1. *Over the next several months, we will interview some of you who take this survey. You will receive $25 and your responses will remain confidential.* ***Would it be ok to contact you for a brief interview after you complete this survey so that we can learn more about your opinions of school meals?*** *(Interviews can be scheduled at a day and time that you choose).* Yes No

2. *Over the next several months, we will be asking some parents for their thoughts about the Universal Meals Program.* ***Are you interested in promoting this survey to families in your SFA (School Food Authority)?*** Yes No

(if Yes to any of 2 questions above)

3. **Your email address:** ____

We will use this email address to contact you to schedule an interview and/or provide you with information to share with families.

Please enter your email address a second time: ____

(only if Yes to interview in a above)

4. **Your phone number:** ____

We will ONLY use this as a backup to contact you about scheduling an interview. Your phone number will not be shared with anyone.

Please enter your phone number a second time: ____

[Domain: district foodservice director]

5. **Your Name:** First, Last *(In case we need to ask any questions about your responses)*

6. **Which of the following most appropriately describes your title?**
   a. School Nutrition Director/Foodservice Director
   b. School Nutrition Supervisor/Manager
   c. Other (write in)

7. **How many years have you worked in this leadership role at your district?**
   a. Less than 1 year
   b. 1-4 years
   c. 5-9 years
   d. 10-14 years
   e. 15-19 years
   f. 20 or more years

8. **How many years have you worked in school foodservice in total (including your current position)?**
   a. Less than 1 year
   b. 1-4 years
   c. 5-9 years
   d. 10-14 years
   e. 15-19 years
   f. 20 or more years

9. **How many years have you worked in other institutional, restaurant or other commercial foodservice (not counting the years above in school foodservice)?**
   a. None
   b. Less than 1 year
   c. 1-4 years
   d. 5-9 years
   e. 10-14 years
   f. 15-19 years
   g. 20 or more years

10. **Full District Name** *(not an abbreviation; if you provide school nutrition services to multiple districts, please provide your **primary district**)*

11. **Your Address** *(where you receive work mail)***:** Street, City, State, Zip

12. **Your District's assigned Child Nutrition and Information Payment System Number _______** *(for accessing descriptive school data so that you do not have to report this on the survey)*

13. **What is the <u>highest</u> grade or year of schooling you completed?**
    a. Less than high school ***(skip Q14)***
    b. High school or GED ***(skip Q14)***
    c. Some college, no degree ***(skip Q14)***
    d. Associate's degree
    e. Bachelor's degree
    f. Master's degree
    g. Graduate credits beyond a Master's degree
    h. Doctorate

14. **Do you have a degree in foods and nutrition, family and consumer sciences, nutrition education, foodservice management, culinary arts, business, or public/school administration? *(asked only if answered Q13 d-h)***
    Yes No

[Domain: COVID waivers]

15. **<u>For the current school year (SY 2021-22),</u> which of the following COVID-19 waivers has your SFA utilized?** Click on the name of each waiver to view a fuller description. *Please select a response for each item.*

a.	Seamless Summer Option to offer free meals to all students	Yes No
b.	Noncongregate Feeding to allow meals to be served and consumed by student off-site	Yes No
c.	Meal Service Time Flexibility to allow meal service outside of standard meal times	Yes No
d.	Parent and Guardian Pick-up to allow parent/guardian meal pick up	Yes No
e.	Community Eligibility to extend CEP application deadlines	Yes No
f.	Area Eligibility changes to FRPM threshold or collection of FRPM applications	Yes No
g.	Meal Pattern to allow flexibility for the federal meal pattern requirements	Yes No
h.	Offer Versus Serve to apply OVS under SFSP rules	Yes No
i.	Monitoring Waivers to allow flexibility in monitoring requirements	Yes No
j.	Foodservice Management to allow flexibility in foodservice management company contracts	Yes No
k.	Local School Wellness Policy Triennial Assessments to extend the deadline for completion	Yes No
l.	Selected Reporting Requirements to waive selected reporting requirement for NSLP/SBP	Yes No

[Domain: nutrition program participation]

16. **Currently (SY 2021-22), to what extent does your SFA participate in or use any of the following?** *Please select a response for each item.*

	None or few schools (<25%)	Some schools (25-50%)	Most or all schools (>50%)	Not sure
a. Reimbursable afterschool snack program				
b. Reimbursable afterschool supper program				
c. Seamless Summer Option (SSO) or Summer Foodservice Program (SFSP) for summer 2022				
d. A la carte sales of reimbursable meal components				
e. Competitive food sales during school hours				
f. Certified to receive additional 7 cents reimbursement per lunch or breakfast				
g. USDA foods				

[Domain: meal preparation & procurement]

17. **Currently (SY 2021-22), who prepares reimbursable school meals for your schools?** (*mark all that apply*)

a. District nutrition services department/central kitchen
b. School sites (do **not** count if schools mostly reheat and serve)
c. Another school district vends the meals
d. A foodservice management company
e. Other (write in)

18. **Currently (SY 2021-22), how many elementary, middle and high schools in your SFA prepare reimbursable school lunch entrees *primarily* using the following methods?** *Please enter a response for each item. Please enter 0 if not used or your SFA has no schools of a particular type.*

	Number of schools by type		
	Elementary schools	Middle schools	High schools
a. Convenience preparation (pre-portioned, heat and serve items, e.g., frozen burrito)			
b. Minimal preparation (primarily involving assembling and portioning, e.g., chef's salad made with pre-cut and pre-cooked ingredients			
c. School-made/scratch or modified scratch preparation (use of raw ingredients, some degree of ingredient preparation and cooking when needed, e.g., spaghetti with scratch-prepared sauce)			

19. **Currently (SY 2021-22), does your SFA participate with other SFAs or another organization in a food-purchasing cooperative**?

a. Yes
b. No

20. **Currently (SY 2021-22), in a typical week, how many days at a typical school do you serve foods that were locally grown or produced in your state**?

a. No days
b. 1 day
c. 2 days
d. 3 days
e. 4 days
f. 5 days
g. Don't know

21. **Currently (SY 2021-22), in a typical week, how many days at a typical school do you serve organic foods**?

h. No days
i. 1 day
j. 2 days
k. 3 days
l. 4 days
m. 5 days
n. Don't know

[Domain: meal service]

22. **Currently (SY 2021-22), do you implement the following strategies for the reimbursable school BREAKFAST in any of your elementary, middle or high schools?** *Please select a response for each item. Mark not applicable (NA) if your SFA does not include any elementary, or middle or high schools.*

	School type		
	**Elementary schools**	**Middle schools**	**High schools**
a. Breakfast in the cafeteria before the bell	Yes No Not applicable	Yes No Not applicable	Yes No Not applicable
b. "Grab and go" option(s) (packaged, carry-away or other non-congregate option)	Yes No Not applicable	Yes No Not applicable	Yes No Not applicable
c. Breakfast after the bell in the classroom or other instructional location (counted as instructional minutes)	Yes No Not applicable	Yes No Not applicable	Yes No Not applicable
d. "Second chance" breakfast (available after school begins or during first period but not served in the classroom)	Yes No Not applicable	Yes No Not applicable	Yes No Not applicable
e. Other strategies to increase breakfast participation (e.g., changes in bus schedules or times for breakfast, leftover breakfast foods made available later in the day) (write in)	Yes No Not applicable	Yes No Not applicable	Yes No Not applicable

23. **Currently (SY 2021-22), do you implement the following strategies for the reimbursable school LUNCH in any of your elementary, middle or high schools?** *Please select a response for each item. Mark not applicable (NA) if your SFA does not include any elementary, or middle or high schools.*

	School type		
	**Elementary schools**	**Middle schools**	**High schools**
a. Recess before lunch	Yes No Not applicable	Yes No Not applicable	Yes No Not applicable
b. "Grab and go" option(s) (packaged, carry-away or other non-congregate option)	Yes No Not applicable	Yes No Not applicable	Yes No Not applicable
c. "Offer-versus-serve" option (students are asked whether or not they want a particular meal item)	Yes No Not applicable	Yes No Not applicable	Yes No Not applicable
d. Option for students to pre-order	Yes No Not applicable	Yes No Not applicable	Yes No Not applicable
e. More than one serving line or food station (include mobile carts, kiosks, service windows, salad bars or other self-serve bars)	Yes No Not applicable	Yes No Not applicable	Yes No Not applicable
f. All meal components are provided on every serving line or food station in the required minimum amounts	Yes No Not applicable	Yes No Not applicable	Yes No Not applicable
g. Students must visit multiple serving lines or food stations that together offer all required meal components (e.g., pasta station, fruit and vegetable bar, and milk cooler)	Yes No Not applicable	Yes No Not applicable	Yes No Not applicable
h. Schools are 'open campus' at *lunch* (some or all students can leave campus to purchase lunch)	Yes No Not applicable	Yes No Not applicable	Yes No Not applicable

[Domain: barriers to meal participation]

24. **Thinking about the students who do NOT regularly eat the reimbursable school meals currently (SY 2021-22), how common are the following barriers for the students you serve?** *Please select a response for each item.*

	**None or very few students**	**Some students**	**Many or most students**	**Don't know/ not sure**
a. Students do not like the taste of the food				
b. Students do not think the food is fresh				
c. Students or parents do not think the food is healthy				
d. Students prefer to eat a la carte options				
e. Students get tired of the options				
f. Do not meet students' cultural or non-medical dietary preferences				
g. Portions are not big enough / not enough food provided				
h. Students prefer to eat meals from home or elsewhere				
i. Students often skip meals (e.g., do not eat any breakfast or lunch)				
j. Students unable to get to school on time for breakfast				
k. Students don't have enough time to get and eat the lunch				

l. Students or parents don't understand that meals are free				
m. Students or parents think only needy kids eat school breakfast and don't want to be thought of that way				
n. Students' friends don't eat the school meals				
o. Other (write in)				

-

[Domain: family communications]

25. **<u>To date in SY 2021-22</u>, how many times have you used the following methods to update students and/or parents about school meals?** *Please select a response for each item.*

	Never	1-3 times	4-6 times	>6 times
a. Posted information and/or menus on a website (count as frequently as your website is substantially updated)				
b. Sent email directly to students and/or parents				
c. Sent text messages to students and/or parents				
d. Called students and/or parents by phone/auto-dialer				
e. Posted information on social media				
f. Distributed hardcopy communications (e.g., flier, posters, or newsletter sent home)				
g. Used the school's public address system for announcements				
h. Made announcements on a public radio station				
i. Submitted press release or participated in other print media activities				
j. Other (write in)				
k. In a language other than English ***(if mark never, skip to Q26_never)***				

26. **Thinking about other languages, besides English…***Please select a response for each item.*

[CA options]	**What languages do some district students/parents use at home?**	**In what languages do you usually communicate with students/parents about school meals?**
Arabic	Yes No Don't know	Yes No
Armenian	Yes No Don't know	Yes No
Cantonese	Yes No Don't know	Yes No
Japanese	Yes No Don't know	Yes No
Korean	Yes No Don't know	Yes No
Mandarin	Yes No Don't know	Yes No
Persian/Farsi	Yes No Don't know	Yes No
Russian	Yes No Don't know	Yes No
Spanish	Yes No Don't know	Yes No
Tagalog	Yes No Don't know	Yes No

Vietnamese	Yes No Don't know	Yes No
Other (write in)	Yes No Don't know	Yes No

26_never.* Thinking about other languages, besides English…** *Please select a response for each item.* ***(only asked 'Never' was selected for Q24j)

[CA options]	**What languages do some district students/parents use at home?**
Arabic	Yes No Don't know
Armenian	Yes No Don't know
Cantonese	Yes No Don't know
Japanese	Yes No Don't know
Korean	Yes No Don't know
Mandarin	Yes No Don't know
Persian/Farsi	Yes No Don't know
Russian	Yes No Don't know
Spanish	Yes No Don't know
Tagalog	Yes No Don't know
Vietnamese	Yes No Don't know
Other (write in)	Yes No Don't know

[Domain: supply chain issues]

27. **Currently (SY 2021-22), how much of a challenge are the following issues for your SFA due to COVID-19 and/or supply chain-related issues?** *Please select a response for each item.*

	**Significant Challenge**	**Moderate Challenge**	**Minimal Challenge**	**Not a Challenge**
a. Costs/financial sustainability of school meal programs				
b. Procuring or receiving the *types* of foods or beverages planned				
c. Procuring or receiving the *quantities* of foods or beverages planned				
d. Procuring or receiving non-food supplies or equipment needed for school meals				
e. Meeting federal meal pattern requirements				
f. Meeting student cultural food preferences				
g. Meeting meal modifications for children with medically-related food and nutrition needs				
h. Adequacy of kitchen equipment				
i. Adequacy of school nutrition services staffing				
j. Meal service modifications or disruptions (e.g., social distancing, classroom meals, need for personal protective equipment/PPE, accommodating distance learners)				
k. Increased meal program participation				
l. Reduced meal program participation				

m. Negative feedback or complaints about school meals from parents or students				

[Domain: free school meals this school year]

***28a.* Before SY 2021-22,** were all of your schools already providing free school meals to all students through CEP or Provision 2 or 3?

a. Yes **(skip Q28)**
b. No

***28.* How much, if any, have the following changed as a result of providing school meals to all students at no charge this year (SY 2021-22)?** *Please select a response for each item.*

	Decreased Greatly	Decreased Slightly	No Effect	Increased Slightly	Increased Greatly
a. Student meal participation					
b. Paperwork/administrative burden					
c. Time in line for students to get meals					
d. Crowding in student dining areas					
e. Stigma for students from families with low income					
f. Ease in collecting Meal Application/Alternative Income Forms from families					
g. Unpaid meal charges/debt					
h. School food waste					
i. School meal packaging/solid waste					
j. Foodservice staffing challenges					
k. Scratch/modified scratch cooking					
l. Use of general funds to support school meal programs					

***29.* Across the following service functions, has equipment been purchased in SY 2021-22 (include if you are planning or in the process of procuring for SY 2022-23) for any kitchens in your SFA in order to serve free school meals to all students?** *Please select a response for each item.*

a. Receiving and storage (e.g., platforms and hand trucks, scales, or walk-in refrigerators/freezers) Yes No
b. Food preparation (e.g., slicers, food processors, utility carts, stainless steel work tables, or combi ovens) Yes No
c. Holding and transportation (e.g., refrigerated or non-refrigerated trucks, hot holding mobile carts, or walk-in coolers [separate from receiving/storage refrigerators]) Yes No
d. Salad or fruit/vegetable bars Yes No
e. Other meal service equipment (e.g., mobile milk coolers, steam table pans or serving portion utensils) Yes No

[Domain: free school meals next school year]

30. **In planning to implement the state's Universal Meals Program in SY 2022-23, how much of a concern, if any, are the following foodservice operations?** *Please select a response for each item.*

	Not a Concern	Mild Concern	Moderate Concern	Serious Concern
a. Costs/financial sustainability of school meal programs				
b. Loss of revenue from paid meals				
c. Loss of revenue from competitive food and beverage sales				
d. Inadequate product or ingredient availability				
e. Difficulty sourcing locally-grown or produced items				
f. Challenges in meeting federal school meal nutrition standards				
g. Challenges in maintaining meal quality and variety				
h. Staffing shortages				
i. Lack of adequate time for staff training				
j. Inadequate kitchen equipment				
k. Inadequate kitchen facility and/or storage space				
l. Increases in per student school meal food waste				
m. Increases in per student school meal packaging/solid waste				
n. Low breakfast participation				
o. High breakfast participation				
p. Low lunch participation				
q. High lunch participation				

31. **In planning to implement the state's Universal Meals Program in SY 2022-23, how much of a concern, if any, are the following cafeteria operations?**
Please select a response for each item.

	Not a Concern	Mild Concern	Moderate Concern	Serious Concern
a. Increased time in line for students to get meals/length of lines				
b. Inadequate meal service space				
c. Inadequate dining space				
d. Not enough time for students to eat				

32. **In planning to implement the state's Universal Meals Program in <u>SY 2022-23</u>, how much of a concern, if any, are the following <u>stakeholder issues</u>?**
Please select a response for each item.

	**Not a Concern**	**Mild Concern**	**Moderate Concern**	**Serious Concern**
a. Increased in student and parent complaints				
b. Challenges in meeting student cultural/ethnic food preferences				
c. Challenges in meeting student food allergies/medical nutrition needs				
d. Difficulty obtaining income information from families				
e. Lack of support from district administration (school board, superintendent)				
f. Lack of support from school administration (principals/vice principals)				
g. Lack of support from nutrition services staff				
h. Lack of support from classroom teachers, school nurses and other school personnel				
i. Lack of support from school or district wellness committees				
j. Lack of financial support from state for the Universal Meals Program beyond SY 2022-23				

33. **In preparation for implementing the state's Universal Meals Program in <u>SY 2022-23</u>, are you planning to do any of the following activities?** *Please select a response for each item.*

	Yes	Maybe	No
a. Conduct a taste test activity with students			
b. Involve students in planning school meal menus			
c. Involve students in naming items offered			
d. Present at a parent group meeting (e.g., PTA/PTO to discuss the school meal program			
e. Conduct or arrange for a partner agency to conduct a nutrition education activity in the classroom			
f. Conduct or arrange for a partner agency to conduct a nutrition education activity in the cafeteria/foodservice area			
g. Host a booth/table at a school event to promote/inform about school meals (e.g., a family night or parent-teacher conference night)			
h. Meet with teachers and custodial staff to explain the school meal program or discuss how program can work in classrooms			
i. Discuss student food allergies with school nurse and/or classroom teachers			
j. Share information about the school meal program with a nutrition advisory council or wellness committee			
k. Meet with an advisory group to plan or assess school meal promotion activities			
l. Invite family members to consume a school meal			
m. Invite community members or partner agencies to plan or promote school meals (e.g., local chefs, farmers, dietitians/nutritionists, Cooperative Extension agents, local sports figures, police officers, firefighters, or other local heroes)			
n. Present information about school meals to a local civic or community service group (e.g., a chamber of commerce, Lions Club, Rotary International, or a similar organization)			
o. Develop a communications plan, implement a school meals promotional campaign, and/or conduct press event			

34. In planning for the state's Universal Meals Program in <u>SY 2022-23</u>, what additional resources and information, if any, do you need? *Please select a response for each item.*

	Not Needed	Needed a Little	Needed a Lot
a. Financial management			
b. Additional facilities and/or equipment			
~~c.~~ Menu planning, meal counting and claiming			
d. Cultural diversity in meal planning			
e. Meeting special dietary needs			
f. Food safety			
g. Communications and marketing to students and parents			
h. Making school meals more appealing to students			
i. Increasing school meal participation			
j. Other (write in)			

[Domain: a la carte]

35. <u>In SY2022-23</u>, does your SFA plan to sell food or beverages on an a la carte basis (including milk)?

a. Yes
b. No ***(skip to Q40a)***

36. <u>In SY2022-23</u>, when does your SFA plan to sell food or beverages on an a la carte basis (including milk)?

a. During breakfast only ***(answer Q37 only and then skip Q38)***
b. During lunch only ***(skip to Q38)***
c. During breakfast and lunch ***(ask both Q37 and Q38)***

37. Is milk the <u>only</u> item you plan to sell on an a la carte basis at breakfast?

a. Yes
b. No

38. Is milk the <u>only</u> item you plan to sell on an a la carte basis at lunch?

a. Yes
b. No

[Domain: finances]

39a. **Prior to the pandemic, in SY 2018-19, did any of your schools participate in the School Breakfast Program?** *Please select a response for each school type.* ***(If 'No' selected for all skip to Q41a)***

Elementary	Yes	No
Middle	Yes	No
High	Yes	No

39b. **Prior to the pandemic, in SY 2018-19, did all students receive free breakfast?** *Please select a response for each school type shown.* ***(Only show school types marked 'Yes' in 38a; If 'Yes' selected for all skip to Q41a)***

Elementary	Yes	No
Middle	Yes	No
High	Yes	No

39. **Prior to the pandemic, in SY 2018-19, what was the average price charged for a reimbursable school BREAKFAST for students who were classified as reduced-price?**

School Type	Average Amount
a. Elementary	$_._ _
b. Middle	$_._ _
c. High	$_._ _

40. **Prior to the pandemic, in SY 2018-19, what was the average price charged for a reimbursable school BREAKFAST for students who paid full price?**

School Type	Average Amount
a. Elementary	$_._ _
b. Middle	$_._ _
c. High	$_._ _

41a. **Prior to the pandemic, in SY 2018-19, did any of your schools participate in the National School Lunch Program?** *Please select a response for each school type.* ***(If 'No' for all skip to Q43)***

Elementary	Yes	No
Middle	Yes	No
High	Yes	No

41b*. Prior to the pandemic, in SY 2018-19, did all students receive free lunch?** *Please select a response for each school type shown.* ***(Only show school types marked 'Yes' in 40a; If 'Yes' for all skip to Q43)

Elementary	Yes	No
Middle	Yes	No
High	Yes	No

41. **<u>Prior to the pandemic, in SY 2018-19</u>, what was the average price charged for a reimbursable school LUNCH for students who were classified as <u>reduced-price</u>?**

School Type	Average Amount
a. Elementary	$_._ _
b. Middle	$_._ _
c. High	$_._ _

42. **<u>Prior to the pandemic, in SY 2018-19</u>, what was the average price charged for a reimbursable school LUNCH for students who paid <u>full price</u>?**

School Type	Average Amount
a. Elementary	$_._ _
b. Middle	$_._ _
c. High	$_._ _

43. **<u>Currently (SY 2021-22)</u>, is the meal reimbursement for breakfast and lunch sufficient for your SFA to cover the full cost of producing meals (including food, labor, supplies, and pandemic costs)?**

a. Breakfast — Yes No Don't serve breakfast Don't know/Not sure ***(skip Q45 if marked any but No)***

b. Lunch — Yes No Don't know/Not sure ***(skip to Q46 if marked any but No for both breakfast and lunch)***

44. **What percent of the full cost of producing a school meal does the reimbursement for breakfast cover?**

a. Less than 25%
b. 25-50%
c. 51-75%
d. 76-99%

45. **What percent of the full cost of producing a school meal does the reimbursement for lunch cover?**

a. Less than 25%
b. 25-50%
c. 51-75%
d. 76-99%

46. **Which of the following factors are driving the deficit for your SFA in SY2021-22?** *(mark all that apply)* ***(Ask only if marked No to breakfast and/or lunch question above)***

a. Food costs
b. School nutrition services labor costs
c. Supply costs
d. Equipment costs
e. Facility costs

f. Storage costs
g. Transportation costs
h. Indirect costs (fringe benefits, accounting, payroll, purchasing, facilities managements and utilities)
i. Increased meal program participation
j. Decreased meal program participation
k. Other (write in)
l. Don't know / not sure

47. What **minimum per meal reimbursement rate** do you think is necessary for your SFA to provide meals that meet all federal nutrition standards **and** appeal to students?
a. Breakfast $_._ _
b. Lunch $_._ _

48. What **minimum per meal reimbursement rate** do you think is necessary for your SFA to provide meals that regularly include fresh, locally-grown produce?
a. Breakfast $_._ _
b. Lunch $_._ _

[Domain: household income verification]

49. **Prior to the pandemic, in SY 2018-19, which of the following methods did your SFA use to determine household income?** (*mark all that apply)*
a. Meal applications
b. Alternative income forms
(If mark a and/or b above ask Q49a)
(If mark a above ask Q49b)
(if mark b above ask Q49c)
c. Direct certification
d. Community eligibility
e. Other (write in)

49a. In SY 2018-19 did you have an online (paperless) option for collecting meal applications and/or alternative income forms?
a. Yes
b. No

49b. In SY 2018-19 what was your estimated return rate for meal applications?
a. ___%
b. Don't know

49c. In SY 2018-19, what was your estimated return rate for alternative income forms?
a. ___%
b. Don't know

50. **<u>In the next school year, SY 2022-23</u>, which of the following methods does your SFA plan to use to determine household income?** (*mark all that apply)*

a. Meal applications
b. Alternative income forms
c. Direct certification
d. Community eligibility
e. Other (write in)

[Domain: school meal numbers]

51. **<u>Currently (SY 2021-22)</u>, how many districts are in your school food authority (SFA)**?

a. ______

52. ***a.*** **Please provide the following information for schools (public or charter) included in your SFA for the <u>current school year (SY 2021-22)</u> .** Elementary schools include <u>transitional kindergarten up to grade 6</u>; Middle or junior high schools include grades 6 up to 9; High schools include grades 9 up to 12. Other grade combinations (e.g., TK-8) and school types (e.g., continuation, alternative, and special education) should be included in the row labeled 'Other'. *Please enter 0 in each column if your SFA does not include schools of any of the 4 types.*

School Type	Total number of schools	Total number of students	Number of schools serving Reimbursable School LUNCH	Number of schools serving Reimbursable School BREAKFAST	Number of LUNCH meals claimed in October *2021 for SY 2021-22*	Number of BREAKFAST meals claimed in October *2021 for SY 2021-22*
**SY 2021-22**						
a. Elementary						
b. Middle						
c. High						
d. Other						

52b. **Please provide the following information for schools (public or charter) included in your SFA for the <u>school year prior to COVID-19 (SY 2018-19)</u>.** Elementary schools include <u>transitional kindergarten up to grade 6</u>; Middle or junior high schools include grades 6 up to 9; High schools include grades 9 up to 12. Other grade combinations (e.g., TK-8) and school types (e.g., continuation, alternative, and special education) should be included in the row labeled 'Other'. *Please enter 0 in each column if your SFA does not include schools of any of the 4 types.*

School Type	Total number of schools	Total number of students	Number of schools serving Reimbursable School LUNCH	Number of schools serving Reimbursable School BREAKFAST	Number of LUNCH meals claimed in October *2018 for SY 2018-19*	Number of BREAKFAST meals claimed in October *2018 for SY 2018-19*	Number of schools participating in the Community Eligibility Provision (CEP), Provision 2 or 3 for LUNCH
**SY 2018-19**							

a. Elementary							
b. Middle							
c. High							
d. Other							***(If not 0 for all, ask next Q)***

53. **When did the first schools (if any) in your SFA begin participating in CEP or Provision 2/3 for school lunch?** (As a reference, CEP first became possible in all states in SY 2014-15) ***(If all responses to Q52b = 0 in last column, skip to Q55)***

a. Before SY 2014-15
b. Between SY 2014-15 and SY 2016-17
c. SY 2018-19 or after

[Domain: benefits/challenges of free school meals through CEP/Provision 2 or 3] ***(asked only if last column of Q52b for any school type is greater than 0)***

54. **<u>Prior to the pandemic</u>, what effect, if any, did providing free school meals to all students through CEP or Provision 2 or 3 have on the following?**

	Decrease Greatly	Decrease Slightly	No Effect	Increase Slightly	Increase Greatly
a. Student meal participation					
b. Paperwork/administrative burden					
c. Time in line for students to get meals					
d. Crowding in student dining areas					
e. Stigma for students from families with low income					
f. Unpaid meal charges/debt					
g. School food waste					
h. School meal packaging/solid waste					
i. Foodservice staffing challenges					

[Domain: closing]

55. **Please share any additional comments you have about the school meals programs.** (write in)

Thank you for sharing your information and expertise!
