# Supplementary material for: Providing School Meals to All Students Free of Charge during the COVID-19 Pandemic and Beyond: Challenges and Benefits Reported by School Foodservice Professionals in California
Source: Nutrients. 2022 Sep 17;14(18):3855. doi: 10.3390/nu14183855 (PMC9500887; doi:10.3390/nu14183855)
Supplement: Supplementary file 1 [file nutrients-14-03855-s001.zip › Supplementary Material.pdf]

Supplementary Table S1. Percent of changes reported while implementing universal school meals in SY2021-22 by California school food authorities (n=360 to 359 depending on missingness of responses; excluded SFAs implementing universal school meals to all schools through CEP or another provision).

Change	Decreased Greatly	Decreased Slightly	No Effect	Increased Slightly	Increased Greatly	n
Student meal participation	1.9	7.5	11.4	33.3	45.8	360
Paperwork/administrative burden	9.9	18.9	26.8	27	17.5	355
Time in line for students to get meals	7.8	7.8	34.2	31.7	18.6	360
Crowding in student dining areas	6.7	2.2	49	25.1	17	359
Stigma for students from families with low income	21	18.7	51.3	2.2	0.8	359
Ease in collecting Meal Application/Alternative Income Forms from families	19.5	12	28.1	19.2	21.2	359
Unpaid meal charges/debt	62.8	3.6	27.8	2.8	3.1	360
School food waste	1.7	12	29.5	38.2	18.7	359
School meal packaging/solid waste	0.9	5.4	26.4	37.7	29.8	353
Foodservice staffing challenges	1.1	1.4	20.6	29.2	47.8	360
Scratch/modified scratch cooking	11.4	13.3	51.3	15	9.1	361
Use of general funds to support school meal programs	12.6	8.9	49.4	20.1	8.7	358

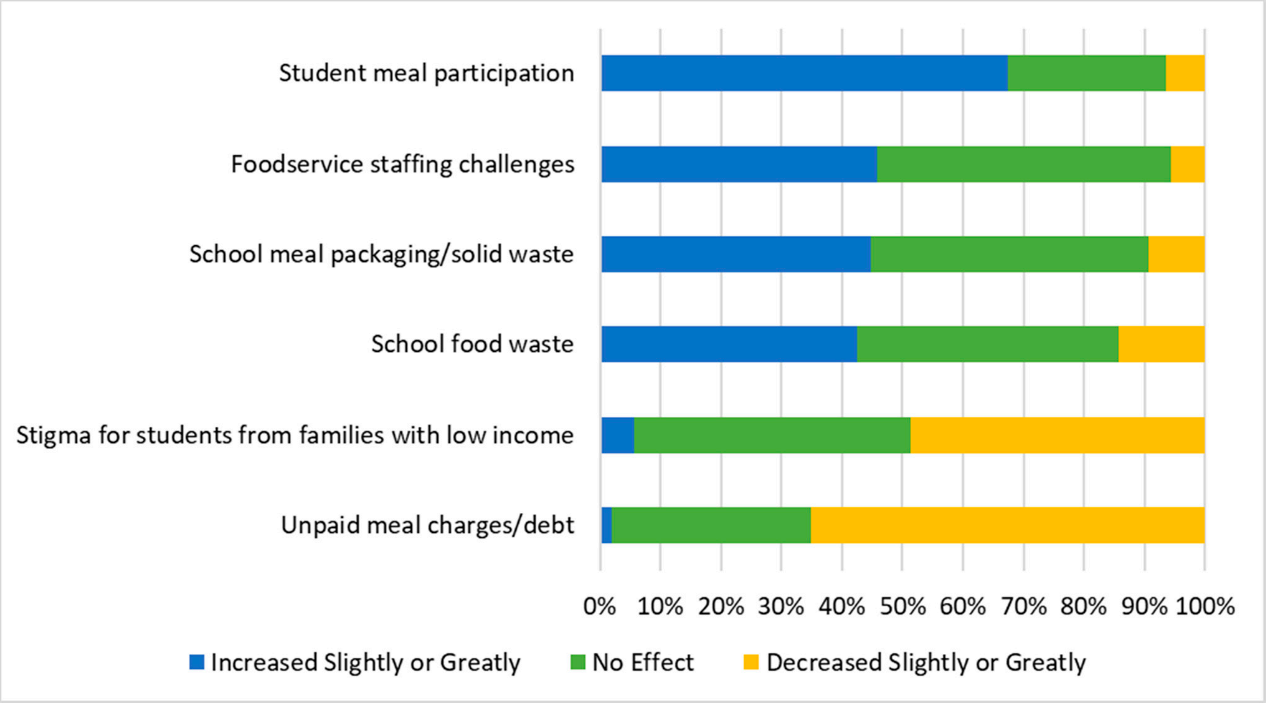

Supplementary Figure S1. Benefits and challenges of USM among SFAs that provided universal school meals before SY2021-22 district-wide (n=106-107 depending on missingness of responses).

Supplementary Table S2. Percent of challenges implementing universal school meals during the COVID-19 pandemic in SY2021-22 by California school food authorities.

Challenge	Significant Challenge	Moderate Challenge	Minimal Challenge	Not a Challenge	n
Costs/financial sustainability of school meal programs	45.6	36.1	11.8	6.6	577
Procuring or receiving the types of foods or beverages planned	64.9	24	7.5	3.7	576
Procuring or receiving the quantities of foods or beverages planned	62.6	23.3	9.2	4.9	575
Procuring or receiving non-food supplies or equipment needed for school meals	62.8	19.8	11.1	6.3	575
Meeting federal meal pattern requirements	19.1	37.2	30.6	13.2	576
Meeting student cultural food preferences	12.4	27.4	38.1	22.2	573
Meeting meal modifications for children with medically-related food and nutrition needs	11.8	21.7	34.8	31.7	575
Adequacy of kitchen equipment	21.4	30.1	26.4	22.1	575
Adequacy of school nutrition services staffing	46.4	26.2	14.4	13	576
Meal service modifications or disruptions	30.1	32.9	25	12	575
Increased meal program participation	20.2	28.4	26.3	25.1	574
Reduced meal program participation	17.6	20.4	22.1	39.9	574
Negative feedback or complaints about school meals from parents or students	9.1	17.3	39.4	34.2	573

Supplementary Table S3. Percent of SFAs that reported that the current meal reimbursement is sufficient to cover the full cost of producing school meals by select characteristics of California school food authorities.

Meal Type[1]	FRPM Eligibility[2]			Urbanicity[3]			Size[4]			
	≤40% (n=145)	>40% (n=215)	p-value		≤40% (n=145)	>40% (n=215)	p-value		≤40% (n=145)	>40% (n=215)
	%			%			%			
Breakfast (n=572)	33.6	44.1	0.07	40.2	43.7	0.54	40.5	48.8	36.8	0.34
Lunch (n=574)	30.1	43.1	0.02	37.5	43.2	0.31	38.7	48.0	34.3	0.23

1. Frequencies represent SFAs that identified the concern as moderate or serious; other answer options were: mild concern and not a concern.
2. Free and reduced-price meal (FRPM) eligibility was defined as SFAs with 40% or less FRPM students vs. SFAs with more than 40% FRPM in SY2021-22. Models for FRPM eligibility were adjusted by CEP status. Adjusted percentages and p-values were reported.
3. Urbanicity was defined using the USDA rural-urban commuting area (RUCA) codes as urban areas=RUCA primary code 1 and non-urban areas=RUCA primary codes 2-10. Models for urbanicity were adjusted by CEP status and size. Adjusted percentages and p-values were reported.
4. SFA size was defined as small=2,499 or less students, medium=2,500 to 9,999 students, and large=10,000 or more students. Models for size were adjusted by CEP status and p-values represent the overall effect of size. Adjusted percentages and p-values were reported.

Supplementary Table S4. Factors reported driving the foodservice deficit for California school food authorities during the COVID-19 pandemic in SY2021-22.

Factors driving the deficit for your SFA in SY2021-22	n	%
Food costs	234	86.4
School nutrition services labor costs	218	80.4
Supply costs	192	70.9
Indirect costs	126	46.5
Equipment costs	122	45.0
Decreased meal program participation	87	32.1
Transportation costs	79	29.2
Facility costs	54	19.9
Storage costs	47	17.3
Increased meal program participation	39	14.4
Other	15	5.5
Don’t know / not sure	7	2.6

Supplementary Table S5. Percent of concerns about implementing universal school meals in SY2022-23 for California school food authorities.

Concern	Not a Concern	Mild Concern	Moderate Concern	Serious Concern	n
Costs/financial sustainability of school meal programs	17.6	25.7	28.5	28.2	575
Loss of revenue from paid meals	47.6	22.7	16.2	13.5	576
Loss of revenue from competitive food and beverage sales	62.2	16.9	13.6	7.3	574
Inadequate product or ingredient availability	12.5	25	26	36.4	576
Difficulty sourcing locally-grown or produced items	19.3	32.4	27.4	20.9	574
Challenges in meeting federal school meal nutrition standards	27.3	28.5	26.6	17.6	575
Challenges in maintaining meal quality and variety	21	29.9	27	22.1	576
Staffing shortages	11.8	19.3	22.6	46.4	576
Lack of adequate time for staff training	20.8	26.6	26.2	26.4	576
Inadequate kitchen equipment	24.7	24.5	30	20.8	576
Inadequate kitchen facility and/or storage space	20.2	22.1	26.1	31.5	574
Increases in per student school meal food waste	18.1	33.6	27	21.4	575
Increases in per student school meal packaging/solid waste	19.7	31	28.2	21.2	575
Low breakfast participation	34.2	29.9	19.6	16.3	576
High breakfast participation	55.5	23.1	14.3	7.1	575
Low lunch participation	39.2	28.7	18.6	13.5	576
High lunch participation	51.4	22.6	16.2	9.9	576

Supplementary Table S6. Percent of additional resources or information needed by California school food authorities to implement universal school meals in SY2022-23.

Resources	Not Needed	Needed a Little	Needed a Lot	n
Financial management	38.5	42.3	19.2	572
Additional facilities and/or equipment	16.2	41.8	42	576
Menu planning, meal counting and claiming	47.8	37.6	14.6	577
Cultural diversity in meal planning	49.6	42.4	8.0	573
Meeting special dietary needs	53.4	37.5	9.1	573
Food safety	62.5	31.9	5.6	571
Communications and marketing to students and parents	23.9	49.1	27	574
Making school meals more appealing to students	28.4	46.3	25.4	575
Increasing school meal participation	28.6	42.7	28.8	574
Other	69.1	7.3	23.6	110
